# Diabetes prevalence and management patterns in US adults, 2001–2023

**DOI:** 10.1007/s00592-025-02572-6

**Published:** 2025-07-30

**Authors:** Miaojin Hu, Michael H. Le, Yee Hui Yeo, Karn Wijarnpreecha, Alisa Likhitsup, Donghee Kim, Vincent L. Chen

**Affiliations:** 1 Department of Biostatistics, University of Michigan, Ann Arbor, MI USA; 2 Robert Larner MD College of Medicine, University of Vermont, Burlington, VT USA; 3 Karsh Division of Gastroenterology and Hepatology, Cedars-Sinai Medical Center, Los Angeles, CA USA; 4 Department of Medicine, Division of Gastroenterology and Hepatology, University of Arizona College of Medicine, Phoenix, AZ USA; 5 Division of Gastroenterology and Hepatology, Department of Internal Medicine, University of Michigan Medical School, Ann Arbor, MI USA; 6 Division of Gastroenterology and Hepatology, Department of Medicine, Stanford University, Stanford, CA USA

**Keywords:** NHANES, Epidemiology, Trends, Care patterns

## Abstract

**Background:**

Diabetes is a leading cause of morbidity and mortality in the United States. We aimed to characterize secular trends in diabetes prevalence, control of glucose and associated comorbidities, and medication use.

**Methods:**

This was a retrospective analysis of National Health and Nutrition Examination Series data from 2001 to 2023. We focused on three outcomes: (1) prevalence of diabetes defined by known diagnosis, hemoglobin A1c ≥ 6.5%, or fasting glucose ≥ 126 mg/dL, and among individuals with diabetes (2) control of glucose levels, low-density lipoprotein, and blood pressure and (3) medication therapy. Predictors were cycle (year) and demographics, specifically age, sex, race/ethnicity, educational level, and household income.

**Results:**

We included 27,437 participants of whom 3467 had diagnosed diabetes and an additional 1602 had undiagnosed diabetes. Diabetes prevalence increased from 10.0% in 2001–2002 to 15.1% in 2021–2023 and was higher in men versus women, and Hispanic/Latino or non-Hispanic Black vs. non-Hispanic White participants. Glycemic control declined over time, from 61.5 to 44.9% having hemoglobin A1c < 7% in 2001–2002 vs. 2021–2023; control was lower in younger participants and those with lower educational attainment. Lipid control improved over time but remained poor: 73.1% had low-density lipoprotein ≥ 70 mg/dL in 2017–2020, and only half of these individuals were taking a statin. There were no significant changes over time in blood pressure control, with 44–56% having blood pressure ≥ 130/80 mmHg. Other than lower lipid control in women, we did not observe differences in control of lipids or blood pressure, or in medication treatment, based on sex and race/ethnicity.

**Discussion:**

Diabetes has increased in prevalence from 2001 to 2023 and management of hyperglycemia and associated risk factors remains inadequate.

**Supplementary Information:**

The online version contains supplementary material available at 10.1007/s00592-025-02572-6.

## Introduction

Diabetes mellitus (DM) is estimated to affect 12–15% of United States (US) adults [1–3] and was the 8th leading cause of death in the US in 2020–2021 [4, 5]. In 2022, DM was associated with > $400 billion in direct and indirect costs [6]. DM is a major risk factor for diseases of the cardiovascular system, kidneys, liver, and other organ systems, and is also associated with increased risk of cancers including of the liver, colon and rectum, pancreas, and breast [7–9].

The last two decades have seen major changes with the potential to impact DM prevalence and care. Risk factors for DM including overweight/obesity and sedentary lifestyle are increasing in prevalence [10–12]. However, treatment options for DM are expanding, with glucagon-like peptide 1 receptor agonists (GLP1RA) and sodium-glucose cotransporter-2 inhibitors (SGLT2i) first approved in the US for DM treatment in 2005 and 2014, respectively [13, 14]. Subsequently, the GLP1RA semaglutide was approved for treatment of overweight/obesity and to decrease the risk of cardiovascular disease [15], while SGLT2i are approved for chronic kidney disease and congestive heart failure associated with T2DM [13, 16]. These medications are more expensive than older drugs, and use of GLP1RA and SGLT2i in diabetes has been reported to be lower in older adults and possibly in Black, Native American, and lower income individuals [17–19]; however, this has not been studied to our knowledge in a US population-based setting. Another crucial aspect of diabetes care which has not been recently characterized in the US population is control of low-density lipoprotein (LDL) [20] and blood pressure [21] to reduce risk of major cardiovascular events which are the leading cause of death in diabetes [22].

Here, we aimed to characterize (1) secular trends in diabetes prevalence, glycemic control, and control of lipids and blood pressure, and (2) differences thereof based on sex, race/ethnicity, and socioeconomic factors.

## Methods

### Study population and design

NHANES is an ongoing survey run by the Centers of Disease Control and Prevention designed to represent the non-institutionalized civilian US population with results released in cycles. In brief, NHANES randomly selects participants throughout the US using a multistage clustered design with unequal probability of selection, starting with primary sampling units (usually a county), followed by secondary sampling units, then individual dwellings within each sampling unit, then sample participants within each unit, with different probability of selecting each individual based on demographics [23]. We included the ten cycles between 2000–2001 and 2021–2023. (Note that the 2017–2020 and 2021–2023 cycles included 4 and 3 years respectively due to field operation changes due to the COVID-19 pandemic [24].) Inclusion criteria were adults (18 years or older) with available data on diabetes diagnosis, fasting glucose, and hemoglobin A1C (HBA1C). Some downstream analyses were performed only in individuals with diabetes.

All analyses incorporated NHANES survey weights to generate estimates that are nationally representative; weights are computed based on the survey design, non-response, and post-stratification. Following the "least common denominator" approach recommended by NHANES reporting guidelines [24–26], fasting subsample weights were used to ensure consistency across analyses (i.e. so that all participants had a minimum amount of data on diabetes diagnosis and glycemic control) while allowing the study population to still be nationally representative. We assessed self-reported diabetes status and distribution of fasting glucose and HBA1C in individuals with missing diabetes questionnaires, fasting glucose, or fasting HBA1C (Supp. Table 1).

### Outcomes

This study focused on three outcomes: (1) prevalence of diabetes, (2) control of glucose levels and associated cardiometabolic risk factors among individuals with diabetes, and (3) medication therapy of individuals with diabetes.

For (1), we defined diabetes as either diagnosed or undiagnosed. Diagnosed diabetes was defined as a self-reported history of diabetes diagnosed by a health professional. Undiagnosed diabetes was defined as not meeting criteria for diagnosed diabetes, but having fasting glucose level ≥ 126 mg/dL or HBA1C ≥ 6.5%. All diabetes included both diagnosed and undiagnosed cases.

For (2), cutoffs of adequate control were defined based on American Diabetes Association 2025 Standards of Care in Diabetes [27–29]. Glycemic control was defined as HBA1C < 7% for the primary analysis and HBA1C < 8% as an alternative threshold. Inadequate control of LDL was defined as LDL ≥ 70 mg/dL in the primary analysis; in a sensitivity analysis, we also assessed those with LDL ≥ 70 mg/dL who did not report taking a statin. Inadequate control of blood pressure was defined as systolic blood pressure (SBP) ≥ 130 mmHg or diastolic blood pressure (DBP) ≥ 80 mmHg; blood pressure was measured three times using an automatic cuff.

For (3), medication use was defined by patient self-report of prescription medications taken in the past 30 days. Diabetes medications were classified as metformin, sulfonylureas, SGLT2i, GLP1RA, insulin, thiazolidinediones, dipeptidyl peptidase-4 inhibitors, or other drugs (alpha-glucosidase inhibitors, bile acid sequestrants, or meglitinides).

### Covariates

Primary correlates of the outcomes included cycle year and demographics, specifically age, sex, race/ethnicity, educational level, and household income. Demographic and health-related data were collected through household interviews.

### Statistical analysis

Descriptive statistics were used to summarize baseline characteristics of the study population, with group differences assessed using Pearson’s chi-square test with Rao and Scott adjustment for categorical variables and the design-based Kruskal–Wallis test for continuous variables. Secular trends were evaluated with joinpoint models using Joinpoint desktop version 5.3.0 [30] (freely available at https://surveillance.cancer.gov/joinpoint/) with settings of a maximum of four joinpoints, uncorrelated errors, model fitting with the Grid Search Method, and model selection based on the weighted Bayesian information criterion; 95% confidence intervals for joinpoint analyses were calculated using empirical quantiles.

Clinical characteristics associated with glycemic control, inadequate BP control, and inadequate LDL control were examined among adults with diagnosed diabetes (or in a sensitivity analysis with diagnosed or undiagnosed diabetes), using Pearson’s chi-square test with Rao and Scott adjustment for categorical variables and the design-based Kruskal–Wallis test for continuous variables. Secular trends in glycemic control and inadequate BP control were assessed in all cycles (2001–2002 through 2021–2023) but trends in LDL control were assessed only 2001–2002 through 2017–2020 (LDL data were not available in the 2021–2023 cycle at the time of analysis). Analyses associating other factors with glycemic or BP control were restricted to 2017–2023, and analyses with inadequate LDL control were limited to the 2017–2020 cycle, to maximize relevance in the present day. Multivariable logistic regression was performed to identify factors associated with inadequate glycemic control, including variables with *p* < 0.1 in unadjusted analyses.

Factors associated with medication use were restricted to the 2017–2020 cycle due to the unavailability of medication data in the 2021–2023 cycle at time of analysis. As above, factors associated with medication use were analyzed using Pearson’s chi-square test with Rao & Scott adjustment for categorical variables and the design-based Kruskal–Wallis test for continuous variables.

Statistical significance was defined as 2-sided *P* < 0.05 throughout. Secular trends and joinpoints were conducted using Joinpoint desktop software as detailed above. All other analyses were performed in R version 4.4.2 utilizing the *survey* package for complex survey design analyses.

## Results

### Cohort

The initial study population included 109,590 participants. After excluding children under 18 years (n = 48,361) and individuals with missing fasting glucose (n = 31,382), hemoglobin A1c (n = 3083), or self-reported diabetes status (n = 35), the final study population included 27,437 participants (Supp. Figure 1). Of these, 3467 had diagnosed diabetes and an additional 1,602 had undiagnosed likely diabetes based on HBA1C ≥ 6.5% or fasting glucose ≥ 126 mg/dl (Table 1). Compared to those without diabetes, participants with either diagnosed and undiagnosed diabetes were older, more often male, and less likely to be college graduates, and had lower income levels (Table 1). Persons with diabetes were more often Hispanic/Latino, non-Hispanic Black, or non-Hispanic Asian than those without diabetes (Table 1). Among those with missing fasting glucose levels (n = 31,382), the unweighted prevalence of diabetes was lower than in the overall cohort (11.1% by HBA1C ≥ 6.5% and 12.2% by self-report) (Supp. Table 1). Similarly, among those with missing HBA1C (n = 3,083), the unweighted prevalence of diabetes was lower than in the overall cohort (11.8% by self-report) (Supp. Table 1). There were too few participants with missing diabetes questionnaires (n = 35), or with missing HBA1C but non-missing fasting glucose (n = 51), for meaningful missingness analysis.

**Table 1:** Clinical characteristics of diagnosed diabetes and undiagnosed diabetes in US adults, 2017–2023

Characteristic	Diagnosed diabetes N = 1,104	Undiagnosed diabetes N = 429	No diabetes N = 5,953	*P* value
Age (years)				< 0.001
18- < 30	14 (2%)	14 (4%)	1163 (24%)	
30- < 40	38 (6%)	38 (8%)	990 (19%)	
40- < 50	100 (12%)	59 (16%)	891 (16%)	
50- < 60	232 (23%)	78 (22%)	899 (16%)	
60- < 70	376 (29%)	126 (27%)	1101 (14%)	
70- < 85	344 (28%)	114 (23%)	909 (11%)	
Sex				0.005
Male	589 (56%)	217 (49%)	2702 (48%)	
Female	515 (44%)	212 (51%)	3251 (52%)	
Education (N = 7196)				< 0.001
No high school	123 (7%)	45 (8%)	305 (3%)	
Some high school but no diploma	154 (11%)	56 (9%)	485 (6%)	
High school diploma or general Educational Development	266 (31%)	105 (28%)	1,244 (26%)	
Some college or associate degree	340 (28%)	120 (30%)	1,797 (30%)	
Bachelor degree or above	219 (23%)	99 (25%)	1,838 (35%)	
Race/ethnicity				0.008
Mexican American	147 (11%)	48 (10%)	594 (9%)	
Other Hispanic	124 (8%)	51 (8%)	599 (8%)	
Non-Hispanic White	432 (55%)	163 (53%)	2,773 (62%)	
Non-Hispanic Black	235 (14%)	96 (16%)	1,094 (11%)	
Non-Hispanic Asian	70 (6%)	17 (4%)	338 (5%)	
Other	96 (6%)	54 (8%)	555 (6%)	
Birth country (n = 7484)				0.019
United States	788 (79%)	283 (73%)	4,473 (80%)	
Other	316 (21%)	146 (27%)	1,478 (20%)	
Smoking status (n = 7,748)				< 0.001
Never smoker	538 (49%)	243 (59%)	3,685 (62%)	
Former smoker	385 (35%)	125 (27%)	1,332 (23%)	
Current smoker	178 (16%)	61 (14%)	931 (14%)	
Insured (n = 7,469)				< 0.001
No	80 (5.5%)	53 (15%)	790 (12%)	
Yes	1,023 (95%)	374 (85%)	5,149 (88%)	
Insurance status (n = 6,442)				< 0.001
Medicaid	102 (9%)	37 (10%)	629 (10%)	
Medicare	340 (28%)	106 (24%)	751 (10%)	
Other	81 (8%)	32 (9%)	476 (9%)	
Private	488 (56%)	194 (57%)	3,206 (71%)	
Ratio of family income to poverty (n = 6,481)				0.007
< 1	175 (14%)	68 (13%)	886 (13%)	
1- < 2	280 (25%)	105 (23%)	1,124 (18%)	
≥ 2	483 (61%)	200 (64%)	3,160 (69%)	
Vital signs and laboratory values				
Fasting glucose (mg/dL)	140 (119, 186)	132 (127, 146)	99 (94, 106)	< 0.001
Hemoglobin A1c (%)	7.00 (6.30, 8.10)	6.50 (6.00, 6.80)	5.40 (5.10, 5.60)	< 0.001
Body mass index (kg/m^2^) (n = 7,368)	32 (28, 37)	33 (29, 38)	28 (24, 32)	< 0.001
Waist circumference (cm) (n = 7,143)	110 (101, 122)	111 (101, 124)	96 (86, 108)	< 0.001
Low-density lipoprotein (mg/dL) (n = 4,057)	87 (68, 115)	109 (90, 133)	108 (88, 131)	< 0.001
High-density lipoprotein (mg/dL) (n = 7,299)	45 (39, 54)	42 (39, 54)	53 (44, 64)	< 0.001
Triglycerides (mg/dL) (n = 4,089)	126 (90, 175)	119 (87, 172)	82 (57, 124)	< 0.001
Systolic blood pressure (mmHg) (n = 6,964)	124 (114, 140)	125 (117, 137)	117 (109, 128)	< 0.001
Diastolic blood pressure (mmHg) (n = 6,964)	74 (66, 82)	77 (69, 86)	73 (66, 80)	< 0.001
Aspartate aminotransferase (U/L) (n = 4,056)	18 (15, 24)	20 (16, 26)	19 (16, 23)	0.142
Alanine aminotransferase (U/L) (n = 4,071)	19 (14, 29)	22 (15, 31)	17 (13, 25)	0.004

Data presented are only for the 2017–2020 and 2021–2023 cycles, not the entire study population. The poverty cutoff is nationally-determined by the US Census Bureau each year and depends on household size (https://www.census.gov/data/tables/time-series/demo/income-poverty/historical-poverty-thresholds.html). Variables are presented as median (interquartile range) or N (%)

### Secular trends in diabetes prevalence

**Fig. 1:**
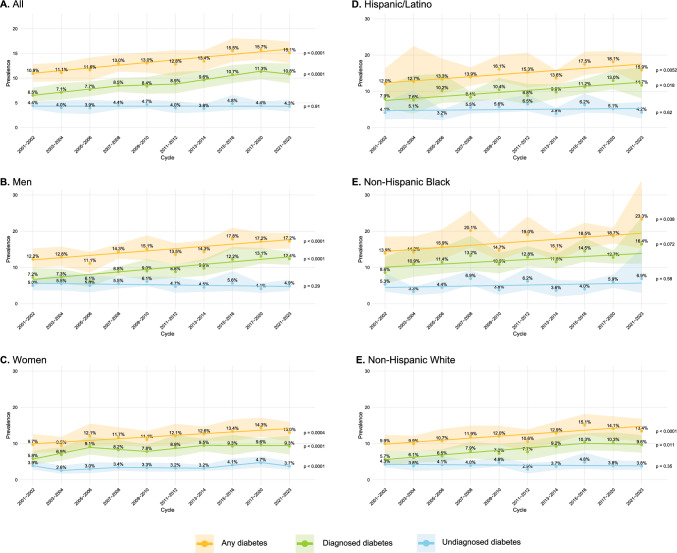
Secular trends in diabetes prevalence in the United States. Prevalence is shown from 2001 to 2002 through 2021–2023 in **A** all participants, **B** men, **C** women, **D** Hispanic/Latino participants, **E** non-Hispanic Black participants, and **F** non-Hispanic White participants. Values are shown as weighted prevalence. Diagnosed diabetes is defined by patient self-report. Undiagnosed diabetes is defined as hemoglobin A1c ≥ 6.5% or fasting glucose > 125 mg/dL but no patient self-report. All diabetes is defined as either diagnosed or undiagnosed diabetes. *P* values are based on timepoint analysis set with no joinpoints to identify the overall trend

In 2021–2023, the weighted prevalence of all diabetes was 15.1% (95% confidence interval [CI] 13.1–17.4%) overall. Between 2001–2023, prevalence of all diabetes increased over time, with annual percentage change (APC) 4.2% (94% CI 2.7–5.9%) and no evidence of a joinpoint (*p* < 0.001) (Fig. 1, Supp. Table 3). The weighted prevalence of diagnosed diabetes was 6.5% (95% CI 5.3–8.0%) in 2001–2002 and 10.8% (95% CI: 9.0–13.0%) in 2021–2023 (Fig. 1A); there were four segments of which three showed positive APC (ranging from 2.2 to 9.1%) and one corresponding to 2017–2020 to 2021–2023 showing a negative annual percentage change (− 5.3%) (Supp. Table 3). The prevalence of undiagnosed diabetes did not significantly change during the study period and was 4.4% (95% CI 3.7–5.2%) in 2001–2002 and 4.3% (95% CI 3.3–5.6%) in 2021–2023 (*p* = 0.91) (Fig. 1A).

In men, the prevalence of all diabetes and diagnosed diabetes increased over time with no joinpoints and APC 4.3% (95% CI 2.6–6.6%) and 7.6% (95% CI 4.6–11.3%), respectively; there was no significant change over time in undiagnosed diabetes (Fig. 1B, Supp. Table 3). In women, prevalence of all diabetes increased over time with no joinpoints (APC 4.0%, 95% CI 1.8–6.5%); diagnosed diabetes overall increased slightly with three joinpoints while undiagnosed diabetes was unchanged overall (Supp. Table 3). The 2021–2023 weighted prevalence of all diabetes was lower in women (13.0%) than men (17.2%) (*p* < 0.01). Overall diabetes prevalence increased with no evidence of joinpoints in Hispanic/Latino (Fig. 1D), non-Hispanic Black (Fig. 1E), and non-Hispanic White (Fig. 1F) participants (*p* < 0.05 for all) (Supp. Table 3). In 2021–2023, weighted diabetes prevalence was highest in non-Hispanic Black (23.3%, 95% CI 15.3–33.9%), followed by Hispanic/Latino (15.9%, 95% CI 12.1–20.5%), and then in non-Hispanic White participants (13.4%, 95% CI 10.6–16.8%) (*p* = 0.048). Prevalence of diagnosed diabetes had an overall significant or borderline significant increase in all race/ethnic groups during at least one period, while undiagnosed diabetes did not change with time in any of the racial/ethnic groups (Supp. Table 3).

### Glycemic control: trends and predictors

**Fig. 2:**
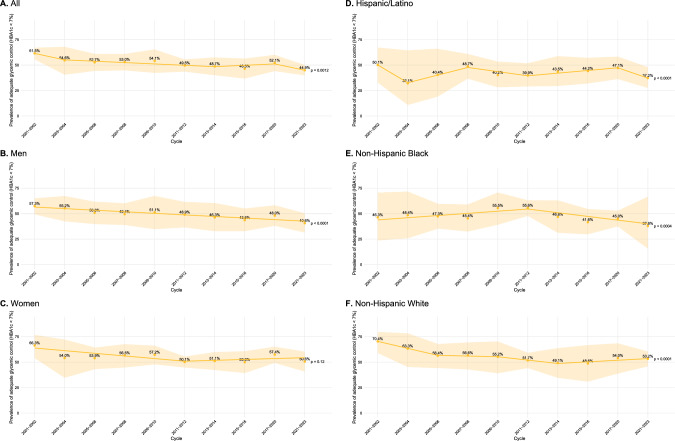
Secular trends in glycemic control among United States adults with diagnosed diabetes. Prevalence is shown from 2001–2002 through 2021–2023 in **A** all participants, **B** men, **C** women, **D** Hispanic/Latino participants, **E** non-Hispanic Black participants, and **F** non-Hispanic White participants. Glycemic control is defined as hemoglobin A1c < 7%. *P* values are based on timepoint analysis set with no joinpoints to identify the overall trend

Among people with diagnosed diabetes, the weighted prevalence of adequate glycemic control, defined as HBA1C < 7%, decreased over time from 61.5% (95% CI 55.6–67.1%) in 2001–2002 overall to 44.9% (95% CI 39.9–50.1%) in 2021–2023 with annual percent change − 2.6% (95% − 4.2% to − 0.9%, p = 0.004); there were 3 joinpoints (Fig. 2A, Supp. Table 4). In the 2017–2023 period, people with adequate glycemic control (vs. inadequate glycemic control) were more often older and had higher educational levels (29% vs. 17% with Bachelor degree or higher) (Table 2). There was a significant association between female sex and adequate control (*p* = 0.029). There were no differences based on race/ethnicity, birth country, or smoking status (Table 2). In the 2017–2023 period, we generated a multivariable logistic regression where glycemic control (HBA1C < 7%) was the outcome, including all predictors associated with glycemic control as *p* < 0.1, adjusted for diabetes treatment status (insulin, oral medication, both, or neither). In this model, increasing age and female sex were associated with higher likelihood of glycemic control (Supp. Table 5). There was a non-significant association between college degree or above and improved glycemic control (*p* = 0.072) (Supp. Table 5).

**Table 2:** Factors associated with adequate glycemic control in diagnosed diabetes

Characteristic	Inadequate control N = 565	Adequate control N = 539	P value
Age (years)			0.021
18- < 30	8 (4%)	6 (1%)	
30- < 40	21 (5%)	17 (6%)	
40- < 50	61 (13%)	39 (11%)	
50- < 60	141 (27%)	91 (19%)	
60- < 70	182 (28%)	194 (29%)	
70- < 85	152 (22%)	192 (34%)	
Sex			0.029
Male	313 (61%)	276 (52%)	
Female	252 (39%)	263 (48%)	
Education (n = 1102)			0.014
No high school	64 (7%)	59 (6%)	
Some high school but no diploma	96 (14%)	58 (9%)	
High school diploma or General Educational Development	142 (32%)	124 (31%)	
Some college or Associate degree	175 (30%)	165 (26%)	
Bachelor degree or above	88 (17%)	131 (29%)	
Race/ethnicity			0.13
Mexican American	87 (14%)	60 (8%)	
Other Hispanic	61 (7%)	63 (9%)	
Non-Hispanic White	200 (50%)	232 (60%)	
Non-Hispanic Black	125 (17%)	110 (12%)	
Non-Hispanic Asian	51 (6%)	45 (6%)	
Other	41 (7%)	29 (6%)	
Birth country			0.57
United States	398 (80%)	390 (78%)	
Other	167 (20%)	149 (22%)	
Smoking status (n = 1101)			0.77
Never smoker	282 (51%)	256 (47%)	
Former smoker	190 (34%)	195 (36%)	
Current smoker	93 (15%)	85 (16%)	
Insured (n = 1,103)			0.18
No	55 (7%)	25 (4%)	
Yes	509 (93%)	514 (96%)	
Insurance status (n = 1011)			0.16
Medicaid	57 (11%)	45 (6%)	
Medicare	159 (28%)	181 (27%)	
Other	43 (7%)	38 (8%)	
Private	243 (54%)	245 (58%)	
Ratio of family income to poverty (n = 938)			0.53
< 1	87 (13%)	88 (16%)	
1- < 2	150 (27%)	130 (23%)	
≥ 2	240 (60%)	243 (62%)	
Fasting glucose (mg/dL)	185 (151, 235)	124 (110, 137)	< 0.001
Hemoglobin A1c (%)	8.00 (7.40, 9.50)	6.30 (6.00, 6.60)	< 0.001
Body mass index (kg/m^2^) (n = 1,073)	32 (28, 37)	32 (27, 37)	0.47
Waist circumference (cm) (n = 1,025)	112 (101, 122)	109 (100, 122)	0.14
Low-density lipoprotein (mg/dL) (n = 633)	87 (62, 117)	88 (70, 114)	0.80
High-density lipoprotein (mg/dL) (n = 1,072)	42 (37, 52)	48 (41, 56)	< 0.001
Triglycerides (mg/dL) (n = 644)	148 (101, 208)	113 (84, 152)	< 0.001
Systolic blood pressure (mmHg) (n = 1,023)	125 (114, 141)	124 (115, 138)	0.96
Diastolic blood pressure (mmHg) (n = 1,023)	74 (67, 82)	73 (65, 81)	0.133
Aspartate aminotransferase (U/L) (n = 638)	18 (15, 24)	18 (14, 24)	0.484
Alanine aminotransferase (U/L) (n = 640)	20 (16, 31)	16 (13, 25)	0.001

Data presented are only for the 2017–2020 and 2021–2023 cycles, not the entire study population. The poverty cutoff is nationally-determined by the US Census Bureau each year and depends on household size (https://www.census.gov/data/tables/time-series/demo/income-poverty/historical-poverty-thresholds.html). Variables are presented as median (interquartile range) or N (%)

Among men with diagnosed diabetes, 57.3% (95%CI 49.3–65.0%) and 40.6% (95% CI 31.5–50.4%) had adequate glycemic control in 2001–2002 and 2021–2023, respectively, with annual percent change − 3.1% (95% CI − 4.5% to − 1.9%, *p* < 0.0001) and no joinpoints (Fig. 2B, Supp. Table 4). There was no trend toward change in glycemic control over time among women (Fig. 2C). In Hispanic/Latino (Fig. 2D) or non-Hispanic Black (Fig. 2E) participants, glycemic control overall worsened from 2001–2002 through 2021–2023 though there were several joinpoints inconsistent with this trend (Supp. Table 4). In non-Hispanic White individuals, glycemic control worsened from 2001–2022 through 2013–2014 (annual percentage change − 5.2%, *p* = 0.0072) then was unchanged from 2013–2014 through 2021–2023 (*p* = 0.25) (Fig. 2F).

We conducted a sensitivity analysis defining adequate glycemic control as HBA1C < 8% rather than < 7% (Supp. Figure 2, Supp. Table 6). As expected a higher proportion of participants attained glycemic control in all subgroups than in the primary analysis. There was a slight decrease in proportion overall with adequate glycemic control in men, Hispanic, and non-Hispanic White participants (Supp. Figure 2, Supp. Table 6). In another sensitivity analysis, we assessed glycemic control among all participants with diabetes, not only diagnosed diabetes. Factors associated with glycemic control were similar, and sex and race/ethnicity were significantly associated with risk of adequate control (Supp. Table 7).

### Control of related comorbidities

Next, we assessed adequacy of control of diabetes-associated comorbidities, specifically lipids and blood pressure. Inadequate blood pressure control was defined as SBP ≥ 130 or DBP ≥ 80 mmHg. Approximately 44–56% of individuals with diagnosed diabetes had inadequate blood pressure control during the study period (Supp. Table 8). There was no significant difference in the proportion of inadequate blood pressure throughout the study period (APC − 0.1%, 95% CI − 3.4% to 3.7% *p* = 0.97). Non-Hispanic Black participants more often had inadequate blood pressure control than other race/ethnic groups (59.8% vs. 49–53%; *p* = 0.012). There were also no significant differences in adequacy of blood pressure control based on sex, country of birth, educational status, insurance type, or income (Supp. Table 8).

Inadequate lipid control was defined as LDL ≥ 70 mg/dL. Over time, the percentage of people with inadequate lipid control declined: 94.3% (95% CI 91.6 to 98.4%) in 2001–2002 vs. 73.1% (95% CI 67.1% to 78.4%) in 2017–2020 (APC − 2.7%, 95% CI − 5.3% to − 0.9%, *p* = 0.0008 with no joinpoints) but remained high (Supp. Table 9). (LDL data are not available for the 2021–2023 cycle.) Women were more likely to have LDL ≥ 70 mg/dL (83.3%, 95% CI 75.6 to 88.9%) than men (65.2%, 95% CI 56.5 to 73.0%) (*p* = 0.0016). There were no significant differences based on race/ethnicity, country of birth, educational attainment, insurance type, or income (Supp. Table 9). We also examined the proportion of individuals with inadequate lipid control who were also not on a statin for LDL lowering (Supp. Table 9). This proportion decreased between 2001–2002 to 2005–2006 (APC − 19.9%, 95% − 35.6 to -12.2%, *p* < 0.0001) with no statistically significant change from 2005–2006 to 2021–2023 (APC − 4.6%, 95% − 7.1 to 2.1%, *p* = 0.10) (Supp. Table 10). The association between sex and lipid control was no longer significant in this analysis; findings were otherwise concordant.

### Diabetes treatment

We evaluated self-reported intake of key drug classes for diabetes treatment from 2001–2002 through the 2017–2020 cycle (data were not available for 2021–2023) in people with diagnosed diabetes (Table 3, Supp. Table 10). Between 2001–2002 to 2017–2020, the proportion of participants receiving treatment increased from 68.6% (95% CI 59.6 to 76.4%) to 87.5% (95% CI 82.5 to 91.2%), respectively; there was one joinpoint and both segments had significantly positive APC (*p* < 0.05) (Supp. Table 10). The mostly widely used medication was metformin, use of which increased from 40.9% (95% CI 34.4 to 47.7%) to 68.1% (95% CI 61.3 to 74.3%) from 2001–2002 to 2017–2020; APC 8.2% (95% CI 6.6 to 10.3%, *p* < 0.0001, no joinpoints). Insulin use also increased from 2001–2002 to 2005–2006 (*p* = 0.027) with no change from 2005–2006 to 2017–2020 (*p* = 0.57) (Supp. Table 10). In contrast, use of sulfonylureas decreased consistently from 42.2% (95% CI 33.3 to 51.5%) to 24.6% (95% CI 20.1 to 29.7%) during this same time period (*p* = 0.022, no joinpoint). Use of thiazolidinediones (which were first approved in 1999 in the US) was stable from 2001–2022 to 2005–2006 (*p* = 0.25) then decreased from 2005–2006 to 2017–2020 (*p* = 0.0028). Use of GLP1RA and SGLT2i increased during the later portion of the study period, though only 4.8% (95% CI 3.2 to 7.1%) and 6.4% (95% CI3.5 to 11.4%) reported GLP1RA or SGLT2i use, respectively, in 2017–2020. The pattern was overall similar when limited to those with diagnosed diabetes who presumably had an indication for treatment, i.e. HBA1C ≥ 7% or already on treatment (Supp. Tables 11 and 12).

**Table 3:** Secular trends in diabetes treatment among diagnosed diabetes

Cycle	2001–2002	2003–2004	2005–2006	2007–2008	2009–2010	2011–2012	2013–2014	2015–2016	2017–2020	*P* value
Any	166/222 (68.6%)	177/234 (73.3%)	179/223 (81.2%)	280/340 (83.5%)	267/327 (82.4%)	282/332 (85.3%)	251/303 (83.3%)	315/382 (89.1%)	566/655 (87.5%)	2001–2002 to 2017–2020: *p* < 0.0001
Metformin	72/222 (40.9%)	102/234 (51.7%)	96/223 (48.2%)	167/340 (49.%)	173/327 (54.4%)	176/332 (55.7%)	172/303 (61.4%)	219/382 (65.9%)	402/655 (68.1%)	2001–2002 to 2017–2020: *p* < 0.0001
Sulfonyl-urea	96/222 (42.2%)	111/234 (44.0%)	82/223 (37.6%)	135/340 (38.5%)	115/327 (36.3%)	113/332 (44.4%)	72/303 (23.1%)	99/382 (22.6%)	156/655 (24.6%)	2001–2002 to 2017–2020: *p* = 0.022
SGLT2i	0/222 (0%)	0/234 (0%)	0/223 (0%)	0/340 (0%)	0/327 (0%)	0/332 (0%)	0/303 (0%)	8/382 (2.4%)	37/655 (6.4%)	2015–2016 to 2017–2020: < 0.0001
GLP1RA	0/222 (0%)	0/234 (0%)	2/223 (2.0%)	1/340 (0.4%)	5/327 (1.8%)	6/332 (2.3%)	11/303 (5.0%)	13/382 (7.0%)	36/655 (4.8%)	2005–2006 to 2015–2016: *p* = 0.038 2015–2016 to 2017–2020: *p* = 0.46
Insulin	39/222 (5.9%)	32/234 (8.2%)	53/223 (22.9%)	74/340 (23.9%)	61/327 (17.6%)	99/332 (30.1%)	93/303 (29.9%)	112/382 (27.2%)	174/655 (21.8%)	2001–2002 to 2005–2006: *p* = 0.046 2005–2006 to 2017–2020: *p* = 0.80
TZD	27/222 (11.9%)	51/234 (25.9%)	47/223 (25.8%)	75/340 (22.2%)	44/327 (18.7%)	24/332 (9.5%)	7/303 (1.6%)	13/382 (3.5%)	24/655 (5.5%)	2001–2002 to 2005–2006: *p* = 0.25 2005–2006 to 2017–2020: *p* = 0.0028
DPP4	0/222 (0%)	0/234 (0%)	0/223 (0%)	16/340 (6.5%)	21/327 (10.2%)	32/332 (13.0%)	28/303 (9.7%)	41/382 (8.9%)	61/655 (13.1%)	2001–2002 to 2017–2020: *p* = 0.38
Other	4/222 (2.9%)	7/234 (3.6%)	2/223 (0.4%)	14/340 (4.5%)	7/327 (2.4%)	8/332 (2.7%)	7/303 (1.0%)	4/382 (1.3%)	11/655 (1.8%)	2001–2002 to 2017–2020: *p* = 0.062

Secular trends in use of medications for treatment of diabetes, among participants with diagnosed diabetes. Values are shown as n/N (%). P values are by a trend test. Medication abbreviations: DPP4, dipeptidyl peptidase-4 inhibitors. GLP1RA, glucagon-like peptide-1 receptor agonists. SGLT2i, sodium-glucose cotransporter-2 inhibitors. TZD, thiazolidinediones. See Methods for details of drug class definitions

Finally, we assessed predictors in diabetes treatment patterns in the 2017–2020 cycle (Supp. Table 13). The only significant predictor was income, with individuals with higher family income more likely to take metformin and less likely to take insulin. There were no significant differences by sex, race/ethnicity, country of birth, educational attainment, or insurance status in overall medication use or use of any specific medication (Supp. Table 13).

## Discussion

Our findings update earlier studies on nationwide trends in control of glucose levels, blood pressure, and LDL [2, 3, 31]. The prevalence of diabetes has gradually increased between 2001–2002 through 2021–2023. Disappointingly, less than 50% of participants in 2021–2023 achieved adequate glycemic control (HBA1C < 7%), and this proportion decreased over time—and there were differences in glycemic control by sex, race/ethnicity, and educational level. While the proportion of patients with inadequate LDL control decreased over time, it remained unacceptably high (> 70% in 2017–2020); in contrast, there was no significant trend in blood pressure control over time, with > 40% of people with diagnosed diabetes having inadequate control at most timepoints. The proportion of people with diagnosed diabetes who are receiving drug therapy has increased over time, though there were medication class trends with increased use of metformin, insulin, GLP1RA, and SGLT2i, and decreased use of thiazolidinediones and sulfonylureas. These findings highlight major limitations in contemporary diabetes care and areas requiring improvement.

The persistently low rates of adequate control of glucose, blood pressure, and LDL are concerning in light of well-described improvement in clinical endpoints with control of comorbidities described from randomized trials. In one meta-analysis of randomized trials, intensive glucose control was associated with 15–17% reduction in risk of coronary artery or heart disease compared to standard care (mean difference in HBA1C of 0.9%) [32]. Another meta-analysis of randomized trials found that each mmol/L (39 mg/dL) reduction in LDL was associated with 9% lower overall mortality, 13% reduction in vascular mortality, and 21% reduction in major vascular events [20]. Similarly, each 10 mmHg reduction in SBP was associated with 13% lower risk of overall mortality and 11–27% lower risk of all cardiovascular events, coronary heart disease, or stroke[21]. These findings are consistent with other studies showing low compliance with American Diabetes Association Standards of Medical Care guidelines [3], despite generally consistent recommendations on glycemic control and blood pressure for the entirety of the study period [33]. Reassuringly, adherence to LDL lowering guidelines has gradually improved over time along with increasingly stringent treatment recommendations [34], suggesting that improved awareness may be showing effects. Further efforts at education and outreach aimed at both patients and healthcare providers, healthcare system quality improvement and workflow development, and improved access to therapy will be required to improve these limitations [35]

Our findings are generally consistent with previous reports of DM prevalence in the US, though specific cutoffs used for blood pressure and lipids have changed over time [2, 3]. We also compare our findings to reports from other countries. Consistent with our findings, one recent worldwide study found a gradual increase in prevalence of diabetes worldwide [36]. They also found generally increasing rates of diabetes treatment in many countries—especially in central/western Europe and Latin America—but unfortunately little to no change in most of sub-Saharan Africa, the Caribbean, Pacific Islands, and south, southeast, and central Asia [36]. Lipid control improved based on one study from China using a nationwide database of outpatients with DM, which found overall reduction in age-standardized levels of total cholesterol, LDL, and triglycerides from 2009 to 2013 [37], consistent with our findings. In contrast, one study of 11 nationally-representative health surveys in England found that the prevalence of hypertension decreased and control in blood pressure control improved from 2003 to 2019 [38]. Education of medical providers, public awareness, and outreach campaigns must be tailored to individual regions to maximize success.

We observed differences in glycemic control based on sex, race/ethnicity, and socioeconomic status. While we were unable to determine why these differences exist, they may be related to dietary patterns [39], obesity [40, 41], or exercise [42]. While medication access or compliance could also be a contributor, unlike in some other studies [43] while consistent with yet others [18, 19] we did not observe differences in diabetes drug use based on sex or race/ethnicity. This may be due to differences in study population (general population vs. only privately-insured individuals vs. study participants) or reporting: in particular, NHANES collects medication data from only the past 30 days and it is based on self-report rather than e.g. pharmacy data [44, 45], which likely results in underreporting. We did not have medication use data in the 2021–2023 cycle, during which time GLP1RA and SGLT2i use continued to increase [46]. Indeed, the frequency of GLP-1RA and SGLT2i use in our study in the 2017–2020 period is lower than has been reported in some other studies [17, 46].

Strengths of this study include use of a nationally-representative cohort based on complex survey weighting and numerous sensitivity analyses. Limitations include (1) inability to distinguish between type 1 and type 2 diabetes, though we expect that > 90% of the participants with diabetes had type 2 diabetes; (2) lack of institutionalized individuals or those in the military in NHANES; and (3) limited medication data as described in the previous paragraph. The number of participants with diabetes is relatively small (around 3000 participants) and self-reported diabetes status and other variables such as socioeconomic status might be subject to diagnosis, recall, and social desirability bias. A large number of participants had missing fasting glucose or HBA1C values and these individuals appeared to have lower prevalence of diabetes than those with non-missing data, so our estimates may inflate true disease prevalence.

In conclusion, we highlighted trends in prevalence, control of glucose concentrations and comorbidities, and pharmacologic treatment of diabetes in the US. Fewer than half of people diagnosed with diabetes had adequate glycemic control, and > 40% and > 70% of participants with diagnosed diabetes have inadequate blood pressure and lipid control, respectively. Improvements in control of glucose, blood pressure, and lipids are required to reduce morbidity and mortality from diabetes complications.

## Supplementary Information

Below is the link to the electronic supplementary material.

Supplementary file1 (DOCX 265 KB)

## Data Availability

Variable field definitions are shown in Supp. Table 2. Code and cleaned datafiles are available at https://github.com/vincentlchen/NHANES_DM.

## Abbreviations

APC: Annual percentage change
DBP: Diastolic blood pressure
DM: Diabetes mellitus
GLP1RA: Glucagon-like peptide-1 receptor agonist
LDL: Low density lipoprotein
SBP: Systolic blood pressure
SGLT2i: Sodium-glucose cotransporter-2 inhibitor
