# Supplementary material for: Diabetes prevalence and management patterns in US adults, 2001–2023
Source: Acta Diabetol. 2025 Jul 30;62(12):2159–70. doi: 10.1007/s00592-025-02572-6 (PMC12727772; doi:10.1007/s00592-025-02572-6)

**Supp. Table 1**: Missing variable analysis. Summary of self-reported diabetes, fasting glucose levels, and hemoglobin A1c in participants missing one of these variables. Values are reported as median (interquartile range) or n/N (%).

| Trait | Number of non-missing values | Value |  |
| --- | --- | --- | --- |
| Missing fasting glucose (n=31,382) | | | |
| Hemoglobin A1C ≥ 6.5% | 28,350 | 3,137/28,350 (11.1%) |  |
| Self-reported diabetes | 31,361 | 3,816/31,361 (12.2%) |  |
| Missing hemoglobin A1C (n=3,083) | | | |
| Fasting glucose ≥ 126 mg/dL | 51 | 6/51 (11.8%) |  |
| Self-reported diabetes | 3,083 | 364/3,083 (11.8%) |  |
| Missing diabetes questionnaire (n=35) | | | |
| Fasting glucose ≥ 126 mg/dL | 14 | 4/14 (28.6%) |  |
| Hemoglobin A1C ≥ 6.5% | 35 | 4/35 (11.4%) |  |

**Supp. Table 2**: Variable definitions. NHANES, National Health And Nutrition Examination Series; data can be downloaded at <https://www.cdc.gov/nchs/nhanes/>.

| Variable | Cycle | NHANES variable name |
| --- | --- | --- |
| Cycle | All | SDDSRVYR |
| Weight (by fasting lab) | 2001-2017, 2021-2023 | WTSAF2YR |
|  | 2017-2020 | WTSAFPRP |
| Sequence ID | 2001-2023 | SEQN |
| Diagnosed diabetes | 2001-2023 | DIQ010 |
| Taking insulin | 2001-2023 | DIQ050 |
| Taking oral medication for diabetes | 2001-2004, 2009-2023 | DIQ070 |
|  | 2005-2008 | DID070 |
| Demographic | | |
| Age | 2001-2023 | RIDAGEYR |
| Race/ethnicity | 2001-2023 | RIDRETH1 |
|  | 2001-2023 | RIDRETH3 |
| Sex | 2001-2023 | RIAGENDR |
| Country of birth | 2001-2006 | DMDBORN |
|  | 2007-2010 | DMDBORN2 |
|  | 2011-2023 | DMDBORN4 |
| Education level | 2001-2023 | DMDEDUC2 |
| Household income | 2001-2006 | INDHHINC |
|  | 2007-2016 | INDHHIN2 |
| Ratio of family income to poverty | 2001-2023 | INDFMPIR |
| Examination | | |
| Systolic blood pressure | 2001-2016 | BPXSY1-4 |
|  | 2017-2023 | BPXOSY1-3 |
| Diastolic blood pressure | 2001-2016 | BPXDI1-4 |
|  | 2017-2023 | BPXODI1-3 |
| Weight | 2001-2023 | BMXWT |
| Height | 2001-2023 | BMXHT |
| Body mass index | 2001-2023 | BMXBMI |
| Waist circumference | 2001-2023 | BMXWAIST |
| Laboratory | | |
| High density lipoprotein cholesterol | 2001-2002 | LBDHDL |
|  | 2003-2004 | LBXHDD |
|  | 2005-2023 | LBDHDD |
| Low density lipoprotein cholesterol | 2001-2020 | LBDLDL |
| Triglycerides | 2001-2020 | LBXTR |
| Hemoglobin A1c | 2001-2023 | LBXGH |
| Fasting glucose | 2001-2023 | LBXGLU |
| Alanine aminotransferase | 2001-2020 | LBXSATSI |
| Aspartate aminotransferase | 2001-2020 | LBXSASSI |
| Insured | 2001-2004 | HID010 |
|  | 2005-2023 | HIQ011 |
| Insurance type | 2001-2004 | HID030A-E |
|  | 2005-2016 | HIQ031A-J |
|  | 2017-2023 | HIQ032A-I |
| Smoking | | |
| Ever smoker | 2001-2023 | SMQ020 |
| Active smoker | 2001-2023 | SMQ040 |
| Prescription medications | | |
| Taken prescription medicine/past month | 2001-2002 | RXD030 |
|  | 2003-2020 | RXDUSE |
|  | 2021-2023 | RXQ033 |
| Medication name | 2001-2002 | RXD240B |
|  | 2003-2020 | RXDDRUG |
| Insulin | Contains text “insulin” but not “syringe” | |
| GLP1RA | Semaglutide, liraglutide, exenatide, dulaglutide, albiglutide, tirzepatide | |
| SGLT2i | Ends in “gliflozin” | |
| Metformin | Contains text “metformin” | |
| TZD | Ends in “glitazone” | |
| Sulfonylureas | Glyburide, glipizide, glimepiride | |
| GPP4 | DPP4: alogliptin, linagliptin, saxagliptin, sitagliptin | |
| Other | Alpha-glucosidase: acarbose, miglitol  Bile acid sequestrants: colesevelam, cholestyramine  Meglitinides: anything ending in “glinide” | |
| Statin | Rosuvastatin, atorvastatin, simvastatin, pravastatin, lovastatin, fluvastatin, pitavastatin | |

**Supp. Table 3: Joinpoint analysis of diabetes prevalence.** APC, annual percent change. LCL, lower confidence level. UCL, upper confidence interval.

| Overall | | | | | | | | |
| --- | --- | --- | --- | --- | --- | --- | --- | --- |
| Diabetes type | Joinpoints | Segment | Segment Start | Segment End | APC | APC 95% LCL | APC 95% UCL | P-Value |
| All | 0 | 0 | 2001-2002 | 2021-2023 | 4.1907 | 2.664 | 5.864 | <0.0001 |
| Diagnosed | 3 | 0 | 2001-2002 | 2007-2008 | 8.9325 | 7.6899 | 11.7005 | <0.0001 |
| Diagnosed | 3 | 1 | 2007-2008 | 2011-2012 | 2.2426 | 0.332 | 5.0865 | 0.020 |
| Diagnosed | 3 | 2 | 2011-2012 | 2017-2020 | 9.0562 | 7.7738 | 11.1868 | <0.0001 |
| Diagnosed | 3 | 3 | 2017-2020 | 2021-2023 | -5.3269 | -8.8564 | -2.1721 | 0.011 |
| Undiagnosed | 0 | 0 | 2001-2002 | 2021-2023 | -0.0432 | -3.0499 | 3.2959 | 0.91 |
| Men | | | | | | | | |
| Diabetes type | Joinpoints | Segment | Segment Start | Segment End | APC | APC 95% LCL | APC 95% UCL | P-Value |
| All | 0 | 0 | 2001-2002 | 2021-2023 | 4.3119 | 2.588 | 6.6001 | <0.0001 |
| Diagnosed | 0 | 0 | 2001-2002 | 2021-2023 | 7.6234 | 4.6336 | 11.2834 | <0.0001 |
| Undiagnosed | 0 | 0 | 2001-2002 | 2021-2023 | -1.881 | -5.8872 | 2.0735 | 0.29 |
| Women | | | | | | | | |
| Diabetes type | Joinpoints | Segment | Segment Start | Segment End | APC | APC 95% LCL | APC 95% UCL | P-Value |
| All | 0 | 0 | 2001-2002 | 2021-2023 | 3.9987 | 1.8298 | 6.464 | 0.0004 |
| Diagnosed | 3 | 0 | 2001-2002 | 2005-2006 | 25.1897 | 18.5611 | 36.8755 | <0.0001 |
| Diagnosed | 3 | 1 | 2005-2006 | 2009-2010 | -6.4072 | -12.0132 | 1.993 | 0.083 |
| Diagnosed | 3 | 2 | 2009-2010 | 2013-2014 | 10.1752 | 4.6898 | 16.9462 | 0.0072 |
| Diagnosed | 3 | 3 | 2013-2014 | 2021-2023 | -0.1407 | -10.452 | 1.7707 | 0.72 |
| Undiagnosed | 4 | 0 | 2001-2002 | 2003-2004 | -33.5339 | -37.1024 | -29.624 | <0.0001 |
| Undiagnosed | 4 | 1 | 2003-2004 | 2007-2008 | 15.1469 | 11.6434 | 18.0355 | <0.0001 |
| Undiagnosed | 4 | 2 | 2007-2008 | 2013-2014 | -2.1469 | -3.5664 | -0.7311 | 0.0048 |
| Undiagnosed | 4 | 3 | 2013-2014 | 2017-2020 | 22.0747 | 19.5876 | 24.9474 | <0.0001 |
| Undiagnosed | 4 | 4 | 2017-2020 | 2021-2023 | -21.6183 | -25.222 | -17.8113 | <0.0001 |
| Hispanic/Latino | | | | | | | | |
| Diabetes type | Joinpoints | Segment | Segment Start | Segment End | APC | APC 95% LCL | APC 95% UCL | P-Value |
| All | 0 | 0 | 2001-2002 | 2021-2023 | 3.9896 | 1.147 | 7.7496 | 0.0052 |
| Diagnosed | 0 | 0 | 2001-2002 | 2021-2023 | 5.886 | 1.2634 | 11.8771 | 0.018 |
| Undiagnosed | 0 | 0 | 2001-2002 | 2021-2023 | 1.3522 | -6.5839 | 11.7843 | 0.62 |
| Non-Hispanic Black | | | | | | | | |
| Diabetes type | Joinpoints | Segment | Segment Start | Segment End | APC | APC 95% LCL | APC 95% UCL | P-Value |
| All | 0 | 0 | 2001-2002 | 2021-2023 | 3.3834 | 0.2058 | 7.649 | 0.038 |
| Diagnosed | 0 | 0 | 2001-2002 | 2021-2023 | 3.5891 | -0.4111 | 9.3923 | 0.072 |
| Undiagnosed | 0 | 0 | 2001-2002 | 2021-2023 | 2.7357 | -7.6582 | 12.5624 | 0.58 |
| Non-Hispanic White | | | | | | | | |
| Diabetes type | Joinpoints | Segment | Segment Start | Segment End | APC | APC 95% LCL | APC 95% UCL | P-Value |
| All | 0 | 0 | 2001-2002 | 2021-2023 | 4.6139 | 2.7886 | 6.4154 | <0.0001 |
| Diagnosed | 1 | 0 | 2001-2002 | 2015-2016 | 8.7391 | 6.8845 | 21.237 | 0.011 |
| Diagnosed | 1 | 1 | 2015-2016 | 2021-2023 | -1.275 | -15.6992 | 6.5249 | 0.73 |
| Undiagnosed | 0 | 0 | 2001-2002 | 2021-2023 | -1.1134 | -3.828 | 1.326 | 0.35 |

**Supp Table 4: Joinpoint analysis of trends in glycemic control (hemoglobin A1c <7%) among participants with diagnosed diabetes**. APC, annual percent change. LCL, lower confidence level. UCL, upper confidence interval.

| Overall | | | | | | | |
| --- | --- | --- | --- | --- | --- | --- | --- |
| Joinpoints | Segment | Segment Start | Segment End | APC | APC 95% LCL | APC 95% UCL | P-Value |
| 3 | 0 | 2001-2002 | 2003-2004 | -10.3941 | -16.2104 | -3.776 | 0.0012 |
| 3 | 1 | 2001-2002 | 2013-2014 | -2.5449 | -5.8929 | 1.0739 | 0.1056 |
| 3 | 2 | 2013-2014 | 2017-2020 | 2.7191 | -1.8682 | 6.0829 | 0.2276 |
| 3 | 3 | 2017-2020 | 2021-2023 | -12.1034 | -17.2880 | -4.2650 | 0.0020 |
| Men | | | | | | | |
|  | | | | | | | |
| Joinpoints | Segment | Segment Start | Segment End | APC | APC 95% LCL | APC 95% UCL | P-Value |
| 0 | 0 | 2001-2002 | 2021-2023 | -3.0867 | -4.5034 | -1.9130 | <0.0001 |
| Women | | | | | | | |
| Joinpoints | Segment | Segment Start | Segment End | APC | APC 95% LCL | APC 95% UCL | P-Value |
| 1 | 0 | 2001-2002 | 2011-2012 | -4.4255 | -22.9998 | 17.2898 | 0.12 |
| 1 | 1 | 2011-2012 | 2021-2023 | 1.6544 | -26.2356 | 25.6682 | 0.38 |
| Hispanic/Latino | | | | | | | |
| Joinpoints | Segment | Segment Start | Segment End | APC | APC 95% LCL | APC 95% UCL | P-Value |
| 4 | 0 | 2001-2002 | 2003-2004 | -34.8525 | -47.9430 | -25.6408 | <0.0001 |
| 4 | 1 | 2003-2004 | 2007-2008 | 20.8406 | 13.4780 | 35.4721 | <0.0001 |
| 4 | 2 | 2007-2008 | 2011-2012 | -9.0449 | -13.1667 | -4.4549 | <0.0001 |
| 4 | 3 | 2011-2012 | 2017-2020 | 6.2618 | 3.3003 | 13.3911 | <0.0001 |
| 4 | 4 | 2017-2020 | 2021-2023 | -21.4363 | -29.8521 | -9.7119 | <0.0001 |
| Non-Hispanic Black | | | | | | | |
| Joinpoints | Segment | Segment Start | Segment End | APC | APC 95% LCL | APC 95% UCL | P-Value |
| 1 | 0 | 2001-2002 | 2011-2012 | 4.4518 | 0.8063 | 20.0613 | 0.034 |
| 1 | 1 | 2011-2012 | 2021-2023 | -7.4526 | -28.3100 | -3.2579 | 0.0004 |
| Non-Hispanic White | | | | | | | |
| Joinpoints | Segment | Segment Start | Segment End | APC | APC 95% LCL | APC 95% UCL | P-Value |
| 3 | 0 | 2001-2002 | 2005-2006 | -10.2743 | -14.7975 | -8.8485 | <0.0001 |
| 3 | 1 | 2005-2006 | 2009-2010 | -1.1909 | -4.1253 | 0.9108 | 0.26 |
| 3 | 2 | 2009-2010 | 2013-2014 | -6.1861 | -8.5881 | 2.8417 | 0.12 |
| 3 | 3 | 2013-2014 | 2021-2023 | 3.1678 | 0.9161 | 7.6002 | 0.037 |

**Supp Table 5:** Factors associated with adequate glycemic control in people diagnosed with diabetes. Inadequate glycemic control was defined as hemoglobin A1c ≥7%. Effects are shown as odds ratio (95% confidence interval). Adjusted models were adjusted for all factors associated with inadequate glycemic control as p<0.1 in unadjusted analyses, as well as diabetes treatment measured as insulin use, oral medication use, both, or neither.

| Factor | Unadjusted | | Adjusted | |
| --- | --- | --- | --- | --- |
|  | Odds ratio (95% confidence interval) | P value | Odds ratio (95% confidence interval) | P value |
| Age (per year) | 1.02 (1.01, 1.03) | 0.0075 | 1.02 (1.00, 1.04) | 0.034 |
| Female sex (vs. male) | 1.47 (1.02, 2.08) | 0.029 | 1.58 (1.12, 2.23) | 0.011 |
| Race/ethnicity |  |  |  |  |
| Non-Hispanic White | Referent |  | Referent |  |
| Non-Hispanic Black | 0.62 (0.32, 1.19) | 0.15 |  |  |
| Hispanic/Latino | 0.66 (0.40, 1.10) | 0.11 |  |  |
| Other | 0.79 (0.41, 1.53) | 0.48 |  |  |
| Education |  |  |  |  |
| Less than 9th grade | Referent |  | Referent |  |
| Some high school but no diploma or GED | 0.73 (0.34, 1.56) | 0.40 | 0.84 (0.38, 1.83) | 0.65 |
| High school graduate/GED or equivalent | 1.16 (0.60, 2.24) | 0.64 | 1.18 (0.61, 2.31) | 0.61 |
| Some college or AA degree | 1.02 (0.55, 1.88) | 0.95 | 1.02 (0.56, 1.85) | 0.95 |
| College graduate or above | 2.06 (1.06, 4.01) | 0.034 | 1.90 (0.94, 3.83) | 0.072 |
| Non-US born | 1.13 (0.74, 1.70) | 0.57 |  |  |

**Supp Table 6: Joinpoint analysis of trends in glycemic control (hemoglobin A1c <8%) among participants with diagnosed diabetes**. APC, annual percent change. LCL, lower confidence level. UCL, upper confidence interval.

| All | | | | | | | |
| --- | --- | --- | --- | --- | --- | --- | --- |
| Joinpoints | Segment | Segment Start | Segment End | APC | APC 95% LCL | APC 95% UCL | P-Value |
| 2 | 0 | 2001-2002 | 2013-2014 | -3.0393 | -6.5333 | 0.2421 | 0.050 |
| 2 | 1 | 2013-2014 | 2017-2020 | 7.0876 | -5.2812 | 12.9296 | 0.088 |
| 2 | 2 | 2017-2020 | 2021-2023 | -13.8136 | -21.9234 | 0.8431 | 0.062 |
| Men | | | | | | | |
| Joinpoints | Segment | Segment Start | Segment End | APC | APC 95% LCL | APC 95% UCL | P-Value |
| 2 | 0 | 2001-2002 | 2013-2014 | -3.5248 | -7.3482 | -2.3905 | 0.0024 |
| 2 | 1 | 2013-2014 | 2017-2020 | 8.5212 | 2.5645 | 13.6994 | 0.0052 |
| 2 | 2 | 2017-2020 | 2021-2023 | -18.5296 | -26.4674 | -11.1010 | 0.0024 |
| Women | | | | | | | |
| Joinpoints | Segment | Segment Start | Segment End | APC | APC 95% LCL | APC 95% UCL | P-Value |
| 0 | 0 | 2001-2002 | 2021-2023 | -1.0392 | -3.3670 | 1.6192 | 0.42 |
| Hispanic/Latino | | | | | | | |
| Joinpoints | Segment | Segment Start | Segment End | APC | APC 95% LCL | APC 95% UCL | P-Value |
| 4 | 0 | 2001-2002 | 2003-2004 | -22.5906 | -29.9115 | -14.5354 | <0.0001 |
| 4 | 1 | 2003-2004 | 2009-2010 | 4.3138 | 1.9194 | 10.9655 | <0.0001 |
| 4 | 2 | 2009-2010 | 2013-2014 | -8.3940 | -11.3876 | -4.3905 | <0.0001 |
| 4 | 3 | 2013-2014 | 2017-2020 | 9.9151 | 6.7830 | 13.4620 | <0.0001 |
| 4 | 4 | 2017-2020 | 2021-2023 | -14.6492 | -21.7720 | -9.5896 | <0.0001 |
| Non-Hispanic Black | | | | | | | |
| Joinpoints | Segment | Segment Start | Segment End | APC | APC 95% LCL | APC 95% UCL | P-Value |
| 0 | 0 | 2001-2002 | 2021-2023 | -1.7316 | -5.6495 | 2.9787 | 0.46 |
| Non-Hispanic White | | | | | | | |
| Joinpoints | Segment | Segment Start | Segment End | APC | APC 95% LCL | APC 95% UCL | P-Value |
| 1 | 0 | 2001-2002 | 2011-2012 | -4.6386 | -18.2699 | -0.7939 | 0.045 |
| 1 | 1 | 2011-2012 | 2021-2023 | 2.3156 | -1.5361 | 24.5750 | 0.20 |

**Supp. Table 7: Factors associated with adequate glycemic control in all diabetes**. Variables are presented as median (interquartile range) or N (%).

| Characteristic | Inadequate control  N = 661 | Adequate control  N = 872 | P value |
| --- | --- | --- | --- |
| Age (years) | | | 0.048 |
| 18-<30 | 10 (3.3%) | 18 (2.5%) |  |
| 30-<40 | 30 (6.3%) | 46 (6.6%) |  |
| 40-<50 | 80 (15%) | 79 (12%) |  |
| 50-<60 | 165 (27%) | 145 (19%) |  |
| 60-<70 | 205 (27%) | 297 (29%) |  |
| 70-<85 | 171 (21%) | 287 (31%) |  |
| Sex | | | 0.026 |
| Male | 362 (60%) | 444 (50%) |  |
| Female | 299 (40%) | 428 (50%) |  |
| Education (n=1,527) | | | 0.002 |
| No high school | 74 (7.5%) | 94 (6.5%) |  |
| Some high school but no diploma | 108 (14%) | 102 (8.8%) |  |
| High school diploma or General Educational Development | 175 (33%) | 196 (28%) |  |
| Some college or Associate degree | 200 (30%) | 260 (28%) |  |
| Bachelor degree or above | 104 (16%) | 214 (29%) |  |
| Race/ethnicity | | | 0.029 |
| Mexican American | 104 (14%) | 91 (7.8%) |  |
| Other Hispanic | 70 (7.2%) | 105 (8.8%) |  |
| Non-Hispanic White | 228 (49%) | 367 (59%) |  |
| Non-Hispanic Black | 147 (17%) | 184 (13%) |  |
| Non-Hispanic Asian | 67 (6.4%) | 83 (6.1%) |  |
| Other | 45 (6.5%) | 42 (5.2%) |  |
| Birth country | | | 0.74 |
| United States | 455 (78%) | 616 (77%) |  |
| Other | 206 (22%) | 256 (23%) |  |
| Smoking status (n=1,530) |  |  | 0.73 |
| Never smoker | 332 (51%) | 449 (53%) |  |
| Former smoker | 216 (33%) | 294 (33%) |  |
| Current smoker | 113 (16%) | 126 (14%) |  |
| Insured (n=1,530) |  |  | 0.46 |
| No | 74 (9.2%) | 59 (7.5%) |  |
| Yes | 585 (91%) | 812 (92%) |  |
| Insurance status (n=1,380) |  |  | 0.37 |
| Medicaid | 62 (10%) | 77 (8.1%) |  |
| Medicare | 173 (26%) | 273 (27%) |  |
| Other | 55 (9.2%) | 58 (7.0%) |  |
| Private | 287 (54%) | 395 (58%) |  |
| Ratio of family income to poverty (n=1,311) |  |  |  |
| <1 | 101 (13%) | 142 (14%) | 0.497 |
| 1-<2 | 175 (27%) | 210 (22%) |  |
| >=2 | 287 (60%) | 396 (63%) |  |
| Vital signs and laboratory values | | | |
| Fasting glucose (mg/dL) | 183 (150, 235) | 128 (114, 137) | <0.001 |
| Hemoglobin A1c (%) | 8.00 (7.40, 9.50) | 6.30 (5.90, 6.60) | <0.001 |
| Body mass index (kg/m^2^) (n=1,495) | 32 (28, 37) | 32 (28, 37) | 0.82 |
| Waist circumference (cm) (n=1,431) | 112 (101, 122) | 110 (100, 122) | 0.29 |
| Low-density lipoprotein (mg/dL) (n=879) | 90 (64, 124) | 96 (73, 120) | 0.407 |
| High-density lipoprotein (mg/dL) (n=1,491) | 42 (37, 51) | 47 (40, 55) | <0.001 |
| Triglycerides (mg/dL) (n=893) | 140 (101, 208) | 117 (85, 157) | <0.001 |
| Systolic blood pressure (mmHg) (n=1,412) | 125 (115, 140) | 124 (115, 137) | 0.72 |
| Diastolic blood pressure (mmHg) (n=1,412) | 76 (69, 82) | 74 (67, 83) | 0.25 |
| Aspartate aminotransferase (U/L) (n=881) | 19 (15, 25) | 19 (15, 24) | 0.81 |
| Alanine aminotransferase (U/L) (n=885) | 21 (16, 31) | 18 (14, 29) | 0.001 |

**Supp. Table 8: Adequacy of blood pressure control in diagnosed diabetes.** Inadequate blood pressure control was defined as systolic blood pressure ≥ 130 mmHg or diastolic blood pressure ≥ 80 mmHg. Values are reported as n/N (%). All comparisons are by a chi-squared test, except for the secular trend (analysis by cycle) which was by joinpoint analysis.

| DM.doc | Inadequate blood pressure control | P value |
| --- | --- | --- |
| Cycle |  | 0.97 |
| 2001-2002 | 129/222 (55%) |  |
| 2003-2004 | 115/234 (47.2%) |  |
| 2005-2006 | 113/223 (49.4%) |  |
| 2007-2008 | 187/340 (53.7%) |  |
| 2009-2010 | 160/327 (44.2%) |  |
| 2011-2012 | 190/332 (54.8%) |  |
| 2013-2014 | 150/303 (49.8%) |  |
| 2015-2016 | 212/382 (49.9%) |  |
| 2017-2020 | 333/655 (56.4%) |  |
| 2021-2023 | 197/449 (45.8%) |  |
| Sex |  | 0.38 |
| Male | 932/1788 (52.1%) |  |
| Female | 854/1679 (49.9%) |  |
| Race/ethnicity |  | 0.012 |
| Hispanic/Latino | 505/968 (49.1%) |  |
| Non-Hispanic Asian | 89/191 (50.7%) |  |
| Non-Hispanic Black | 494/841 (59.8%) |  |
| Non-Hispanic White | 623/1314 (49.1%) |  |
| Other | 75/153 (53.3%) |  |
| Birth country |  | 0.93 |
| United States | 1306/2524 (51%) |  |
| Other | 479/942 (51.2%) |  |
| Education |  | 0.037 |
| No high school | 318/601 (51.3%) |  |
| Some high school but no diploma | 320/573 (54.2%) |  |
| High school diploma or General Educational Development | 429/793 (56.2%) |  |
| Some college or Associate degree | 437/919 (47.8%) |  |
| Bachelor degree or above | 276/572 (46.7%) |  |
| Insurance status |  | 0.24 |
| Medicaid | 129/273 (45.5%) |  |
| Medicare | 526/959 (54.9%) |  |
| Other | 100/211 (46.8%) |  |
| Private | 840/1614 (51.7%) |  |
| Ratio of family income to poverty |  | 0.16 |
| <1 | 375/694 (54.8%) |  |
| 1-<2 | 469/925 (51.3%) |  |
| >=2 | 740/1462 (48.7%) |  |

**Supp. Table 9: Adequacy of low density lipoprotein control in diagnosed diabetes.** Inadequate low density lipoprotein control was defined as low density lipoprotein level ≥ 70 mg/dL, or low density lipoprotein level ≥ 70 mg/dL and not taking a statin. Values are reported as n/N (%). All comparisons are by a chi-squared test, except for the secular trend (analysis by cycle) which was by joinpoint analysis. For the outcome of low density lipoprotein level ≥ 70 mg/dL, there were no joinpoints and the p value is for the entire period of 2001-2002 to 2017-2020; for the endpoint of low density lipoprotein level ≥ 70 mg/dL and not taking a statin, there was one joinpoint at 2005-2006 with the p value for 2001-2002 being <0.0001 and from 2005-2006 to 2017-2020 being 0.10.

| Predictor | Low density lipoprotein ≥ 70 mg/dL | | Low density lipoprotein ≥ 70 mg/dL and no statin use | |
| --- | --- | --- | --- | --- |
|  | Proportion | P value | Proportion | P value |
| Cycle |  | <0.0001 |  |  |
| 2001-2002 | 134/222 (94.3%) |  | 101/222 (71.3%) | <0.0001 |
| 2003-2004 | 186/234 (88.7%) |  | 125/234 (55.8%) |  |
| 2005-2006 | 173/223 (82.5%) |  | 102/223 (47.8%) |  |
| 2007-2008 | 254/340 (79.1%) |  | 140/340 (44.6%) | 0.10 |
| 2009-2010 | 251/327 (84.8%) |  | 121/327 (38.1%) |  |
| 2011-2012 | 244/332 (74%) |  | 128/332 (38.7%) |  |
| 2013-2014 | 227/303 (81.9%) |  | 113/303 (40.4%) |  |
| 2015-2016 | 273/382 (81.6%) |  | 138/382 (33.3%) |  |
| 2017-2020 | 471/655 (73.1%) |  | 237/655 (36.6%) |  |
| Sex |  |  |  |  |
| Male | 232/347 (65.2%) | 0.0016 | 119/347 (31.8%) | 0.1098 |
| Female | 239/308 (83.3%) |  | 118/308 (42.8%) |  |
| Race/ethnicity |  |  |  |  |
| Hispanic/Latino | 132/179 (77%) | 0.7844 | 68/179 (42.3%) | 0.6346 |
| Non-Hispanic Asian | 49/71 (70.2%) |  | 29/71 (41.1%) |  |
| Non-Hispanic Black | 127/171 (75.8%) |  | 61/171 (38.7%) |  |
| Non-Hispanic White | 135/196 (72%) |  | 64/196 (34.1%) |  |
| Other | 28/38 (68.3%) |  | 15/38 (33.7%) |  |
| Birth country |  |  |  |  |
| United States | 298/434 (70.3%) | 0.0572 | 146/434 (33.8%) | 0.1326 |
| Other | 173/221 (81.9%) |  | 91/221 (45.6%) |  |
| Education |  |  |  |  |
| No high school | 63/87 (74.1%) | 0.2983 | 22/87 (24%) | 0.5254 |
| Some high school but no diploma | 68/94 (81.8%) |  | 38/94 (36.1%) |  |
| High school diploma or General Educational Development | 109/154 (71.7%) |  | 53/154 (35.4%) |  |
| Some college or Associate degree | 148/200 (79%) |  | 78/200 (43.7%) |  |
| Bachelor degree or above | 81/118 (63.6%) |  | 44/118 (34%) |  |
| Insurance status |  | 0.2832 |  | 0.3766 |
| Medicaid | 44/52 (88.9%) |  | 24/52 (45.7%) |  |
| Medicare | 137/193 (76.1%) |  | 52/193 (27.8%) |  |
| Other | 31/49 (66.8%) |  | 17/49 (32.9%) |  |
| Private | 199/294 (68.6%) |  | 104/294 (36%) |  |
| Ratio of family income to poverty |  | 0.1197 |  | 0.1453 |
| <1 | 86/108 (85.3%) |  | 49/108 (49%) |  |
| 1-<2 | 120/182 (69.7%) |  | 55/182 (34%) |  |
| >=2 | 183/263 (70.3%) |  | 91/263 (32.8%) |  |

**Supp. Table 10: Joinpoint analysis of diabetes medication use in diagnosed diabetes**. APC, annual percent change. LCL, lower confidence level. UCL, upper confidence interval. Medication abbreviations: DPP4, dipeptidyl peptidase-4 inhibitors. GLP1RA, glucagon-like peptide-1 receptor agonists. SGLT2i, sodium-glucose cotransporter-2 inhibitors. TZD, thiazolidinediones. See Methods for details of drug class definitions.

| Drug | Joinpoints | Segment | Segment Start | Segment End | APC | APC 95% LCL | APC 95% UCL | P-Value |
| --- | --- | --- | --- | --- | --- | --- | --- | --- |
| Any | 1 | 0 | 2001-2003 | 2005-2006 | 14.4938 | 8.0495 | 27.2909 | 0 |
| Any | 1 | 1 | 2005-2006 | 2017-2020 | 2.4704 | 0.9409 | 3.6334 | 0.020796 |
| DPP4 | 0 | 0 | 2007-2008 | 2017-2020 | 8.3726 | -10.397 | 34.7839 | 0.287143 |
| GLP1RA | 1 | 0 | 2005-2006 | 2015-2016 | 40.3288 | 30.6 | 159.5351 | 0.010398 |
| GLP1RA | 1 | 1 | 2015-2016 | 2017-2020 | -26.0378 | -47.7301 | 20.4074 | 0.360328 |
| Insulin | 1 | 0 | 2001-2002 | 2005-2006 | 126.5976 | 17.1888 | 2830.09 | 0.026795 |
| Insulin | 1 | 1 | 2005-2006 | 2017-2020 | 2.6849 | -10.5003 | 11.7847 | 0.573485 |
| Metformin | 0 | 0 | 2001-2002 | 2017-2020 | 8.1971 | 6.5955 | 10.3352 | 0 |
| SGLT2i | 0 | 0 | 2015-2016 | 2017-2020 | 178.4237 | 178.4237 | 178.4237 | 0 |
| Sulfonylurea | 0 | 0 | 2001-2002 | 2017-2020 | -6.0641 | -12.104 | -1.1382 | 0.021596 |
| TZD | 1 | 0 | 2001-2002 | 2005-2006 | 55.2975 | -11.5019 | 1134.701 | 0.20196 |
| TZD | 1 | 1 | 2005-2006 | 2017-2020 | -30.2187 | -82.5175 | -20.2655 | 0.003599 |
| Other | 0 | 0 | 2001-2002 | 2017-2020 | -10.939 | -21.8123 | -0.9303 | 0.031194 |

**Supp. Table 11: Secular trends in diabetes treatment among people with diabetes and a presumptive indication for treatment**. Secular trends in use of medications for treatment of diabetes, among participants with diabetes with presumptive indication for therapy, defined as hemoglobin A1c ≥ 7% or receiving therapy. Values are shown as n/N (%). P values are by a trend test. Medication abbreviations: DPP4, dipeptidyl peptidase-4 inhibitors. GLP1RA, glucagon-like peptide-1 receptor agonists. SGLT2i, sodium-glucose cotransporter-2 inhibitors. TZD, thiazolidinediones. See Methods for details of drug class definitions.

| Cycle | 2001-2002 | 2003-2004 | 2005-2006 | 2007-2008 | 2009-2010 | 2011-2012 | 2013-2014 | 2015-2016 | 2017-2020 | P value |
| --- | --- | --- | --- | --- | --- | --- | --- | --- | --- | --- |
| Any | 172/213 (77.9%) | 181/217 (87.2%) | 187/228 (82.6%) | 299/346 (89.6%) | 282/345 (81.4%) | 294/346 (85.2%) | 267/306 (88%) | 327/371 (90.4%) | 594/669 (90.7%) | 2001-2002 to 2017-2020: p=0.068 |
| Metformin | 74/213 (45.7%) | 104/217 (60.6%) | 97/228 (47.1%) | 177/346 (52.5%) | 183/345 (53.8%) | 188/346 (56.7%) | 186/306 (65.4%) | 226/371 (66.3%) | 426/669 (71%) | 2001-2002 to 2017-2020: p<0.0001 |
| SU | 99/213 (47.3%) | 114/217 (52.7%) | 85/228 (37.6%) | 144/346 (40.3%) | 121/345 (35.5%) | 114/346 (43%) | 77/306 (24.3%) | 103/371 (22.9%) | 164/669 (25.5%) | 2001-2002 to 2017-2020: p<0.0001 |
| SGLT2i | 0/213 (0%) | 0/217 (0%) | 0/228 (0%) | 0/346 (0%) | 0/345 (0%) | 0/346 (0%) | 0/306 (0%) | 8/371 (2.4%) | 37/669 (6.4%) | 2015-2016 to 2017-2020: p<0.0001 |
| GLP1RA | 0/213 (0%) | 0/217 (0%) | 2/228 (2%) | 2/346 (1.4%) | 5/345 (1.7%) | 6/346 (2.2%) | 11/306 (5%) | 13/371 (7%) | 36/669 (4.8%) | 2005-2006 to 2015-2016: p=0.0060  2015-2016 to 2017-2020: p=0.25 |
| Insulin | 39/213 (6.5%) | 32/217 (9.4%) | 53/228 (22.3%) | 74/346 (23.9%) | 62/345 (16.9%) | 100/346 (29.1%) | 93/306 (30.2%) | 113/371 (27.2%) | 176/669 (22%) | 2001-2002 to 2005-2006: p=0.030  2005-2006 to 2017-2020: p=0.78 |
| TZD | 27/213 (13%) | 51/217 (29.7%) | 49/228 (25.7%) | 80/346 (24.5%) | 45/345 (18.1%) | 26/346 (9.6%) | 9/306 (1.9%) | 14/371 (4.3%) | 25/669 (5.6%) | 2001-2002 to 2007-2008: p=0.62  2007-2008 to 2017-2020: p=0.0024 |
| DPP4 | 0/213 (0%) | 0/217 (0%) | 0/228 (0%) | 16/346 (6.5%) | 23/345 (10.1%) | 33/346 (12.7%) | 30/306 (10%) | 43/371 (8.9%) | 61/669 (13.2%) | 2001-2002 to 2017-2020: p=0.38 |
| Other | 5/213 (3.8%) | 7/217 (4.1%) | 4/228 (2.3%) | 15/346 (4.8%) | 8/345 (2.4%) | 8/346 (2.6%) | 7/306 (1%) | 4/371 (1.3%) | 11/669 (1.8%) | 2001-2002 to 2017-2020: p=0.18 |

**Supp. Table 12: Joinpoint analysis of participants with diagnosed diabetes and presumptive indication for treatment**. Joinpoint analysis of trends in use of medications for treatment of diabetes, among participants with diabetes with presumptive indication for therapy, defined as hemoglobin A1c ≥ 7% or receiving therapy. APC, annual percent change. LCL, lower confidence level. UCL, upper confidence interval. Medication abbreviations: DPP4, dipeptidyl peptidase-4 inhibitors. GLP1RA, glucagon-like peptide-1 receptor agonists. SGLT2i, sodium-glucose cotransporter-2 inhibitors. TZD, thiazolidinediones. See Methods for details of drug class definitions.

| Drug | Joinpoints | Segment | Segment Start | Segment End | APC | APC 95% LCL | APC 95% UCL | P-Value |
| --- | --- | --- | --- | --- | --- | --- | --- | --- |
| Any | 0 | 0 | 2001-2002 | 2017-2020 | 1.1023 | -0.1113 | 2.7257 | 0.068386 |
| DPP4 | 0 | 0 | 2007-2008 | 2017-2020 | 6.6459 | -12.7558 | 31.7746 | 0.389122 |
| GLP1RA | 1 | 0 | 2005-2006 | 2015-2016 | 39.6183 | 30.2178 | 118.3201 | 0.005999 |
| GLP1RA | 1 | 1 | 2015-2016 | 2017-2020 | -27.7769 | -45.8534 | 14.0254 | 0.24915 |
| Insulin | 1 | 0 | 2001-2002 | 2005-2006 | 105.5015 | 11.1081 | 2076.839 | 0.029994 |
| Insulin | 1 | 1 | 2005-2006 | 2017-2020 | 1.3858 | -14.1399 | 10.8841 | 0.784243 |
| Metformin | 0 | 0 | 2001-2002 | 2017-2020 | 5.4093 | 3.0865 | 8.9183 | 0 |
| SGLT2i | 0 | 0 | 2015-2016 | 2017-2020 | 168.876 | 168.876 | 168.876 | 0 |
| Sulfonylurea | 0 | 0 | 2001-2002 | 2017-2020 | -9.6259 | -15.6174 | -5.4781 | 0 |
| TZD | 1 | 0 | 2001-2002 | 2007-2008 | 4.0781 | -18.2064 | 343.1866 | 0.621876 |
| TZD | 1 | 1 | 2007-2008 | 2017-2020 | -35.3207 | -77.1716 | -24.7979 | 0.0024 |
| Other | 0 | 0 | 2001-2002 | 2017-2020 | -14.2712 | -25.9001 | -3.3193 | 0.017197 |

**Supp. Table 13: Predictors of use of medications for diabetes treatment**. P values are by a chi-squared test within each category of interest. Medication abbreviations: DPP4, dipeptidyl peptidase-4 inhibitors. GLP1RA, glucagon-like peptide-1 receptor agonists. SGLT2i, sodium-glucose cotransporter-2 inhibitors. TZD, thiazolidinediones. See Methods for details of drug class definitions.

| Category | Drug class | | | | | | | | |
| --- | --- | --- | --- | --- | --- | --- | --- | --- | --- |
|  | Any | Metformin | Sulfonyl-ureas | SGLT2i | GLP1RA | Insulin | TZD | DPP4 | Other |
| Sex | | | | | | | | | |
| Male | 294/347 (88.1%) | 217/347 (71.5%) | 91/347 (24.8%) | 21/347 (6.0%) | 18/347 (5.2%) | 82/347 (20.4%) | 13/347 (7.3%) | 27/347 (14.9%) | 5/347 (0.6%) |
| Female | 272/308 (86.7%) | 185/308 (63.8%) | 65/308 (24.3%) | 16/308 (7.0%) | 18/308 (4.4%) | 92/308 (23.6%) | 11/308 (3.1%) | 34/308 (10.7%) | 6/308 (3.2%) |
| P value | 0.73 | 0.097 | 0.91 | 0.83 | 0.65 | 0.47 | 0.11 | 0.44 | 0.015 |
| Race/ethnicity | | | | | | | | | |
| Hispanic/Latino | 155/179 (84.7%) | 119/179 (63.9%) | 44/179 (24.0%) | 8/179 (4.5%) | 6/179 (3.9%) | 45/179 (22.6%) | 6/179 (4.5%) | 15/179 (9.3%) | 2/179 (0.5%) |
| Non-Hispanic Asian | 62/71 (88.8%) | 49/71 (70.2%) | 16/71 (25.3%) | 7/71 (9.4%) | 2/71 (1.6%) | 12/71 (14.2%) | 3/71 (3.6%) | 4/71 (4.6%) | 2/71 (2.8%) |
| Non-Hispanic Black | 152/171 (89.8%) | 93/171 (58.0%) | 37/171 (20.9%) | 10/171 (5.2%) | 13/171 (6.6%) | 56/171 (30.5%) | 5/171 (1.9%) | 13/171 (6.9%) | 1/171 (0.8%) |
| Non-Hispanic White | 165/196 (88.1%) | 116/196 (71.4%) | 51/196 (24.9%) | 9/196 (6.8%) | 12/196 (4.9%) | 53/196 (20.9%) | 9/196 (7.2%) | 23/196 (17.0%) | 5/196 (2.3%) |
| Other Race - Including Multi-Racial | 32/38 (84.0%) | 25/38 (70.8%) | 8/38 (31.0%) | 3/38 (8.5%) | 3/38 (6.5%) | 8/38 (17.4%) | 1/38 (1.8%) | 6/38 (10.8%) | 1/38 (1.0%) |
| P value | 0.79 | 0.27 | 0.81 | 0.79 | 0.60 | 0.33 | 0.39 | 0.11 | 0.41 |
| Country of birth | | | | | | | | | |
| United States | 370/434 (86.6%) | 246/434 (65.5%) | 95/434 (21.9%) | 24/434 (6.6%) | 30/434 (6%) | 128/434 (24%) | 17/434 (6.1%) | 42/434 (14.6%) | 7/434 (2%) |
| Others | 196/221 (90.2%) | 156/221 (76.2%) | 61/221 (32.8%) | 13/221 (5.6%) | 6/221 (1.2%) | 46/221 (15.2%) | 7/221 (3.5%) | 19/221 (8.5%) | 4/221 (1.1%) |
| P value | 0.47 | 0.12 | 0.073 | 0.76 | 0.00095 | 0.097 | 0.46 | 0.15 | 0.44 |
| Education | | | | | | | | | |
| No high school | 80/87 (90.8%) | 58/87 (62.8%) | 25/87 (30.8%) | 4/87 (4.1%) | 1/87 (2.2%) | 25/87 (24.5%) | 4/87 (2.7%) | 7/87 (6.7%) | 1/87 (0.6%) |
| Some high school but no diploma | 76/94 (83.4%) | 52/94 (66.7%) | 18/94 (14.5%) | 4/94 (1.6%) | 4/94 (2.2%) | 31/94 (33.7%) | 2/94 (4.7%) | 9/94 (12.6%) | 2/94 (2.4%) |
| High school diploma or General Educational Development | 134/154 (84.7%) | 95/154 (67.8%) | 39/154 (21.9%) | 12/154 (11.1%) | 13/154 (6.1%) | 40/154 (19.2%) | 4/154 (4%) | 16/154 (21.5%) | 1/154 (0.4%) |
| Some college or Associate degree | 172/200 (86.8%) | 118/200 (63.3%) | 51/200 (28.1%) | 9/200 (4.4%) | 12/200 (6.1%) | 56/200 (25.1%) | 7/200 (4.4%) | 18/200 (8.1%) | 3/200 (3.9%) |
| Bachelor degree or above | 102/118 (92.9%) | 78/118 (76.5%) | 23/118 (27.5%) | 8/118 (5.3%) | 6/118 (3.7%) | 21/118 (15%) | 7/118 (10%) | 11/118 (9.7%) | 4/118 (1.2%) |
| P value | 0.12 | 0.33 | 0.31 | 0.18 | 0.52 | 0.20 | 0.52 | 0.15 | 0.17 |
| Insurance status | | | | | | | | | |
| Medicaid | 43/52 (81.8%) | 33/52 (54.1%) | 15/52 (35%) | 5/52 (7.5%) | 3/52 (6.3%) | 14/52 (25.2%) | 1/52 (8.7%) | 5/52 (4.8%) | 0/52 (0%) |
| Medicare | 167/193 (89.2%) | 105/193 (64.9%) | 51/193 (26.3%) | 7/193 (2.7%) | 9/193 (6%) | 65/193 (24.2%) | 5/193 (1.3%) | 19/193 (9.2%) | 3/193 (2.8%) |
| Other | 41/49 (80.9%) | 29/49 (66.3%) | 9/49 (35.1%) | 1/49 (0%) | 2/49 (2.8%) | 12/49 (18.5%) | 2/49 (1.4%) | 1/49 (10.6%) | 2/49 (1.4%) |
| Private | 263/294 (89.8%) | 192/294 (71.6%) | 62/294 (20.6%) | 23/294 (9.4%) | 22/294 (5%) | 70/294 (21.8%) | 14/294 (7%) | 34/294 (17.3%) | 6/294 (1.8%) |
| P value | 0.48 | 0.35 | 0.23 | 0.14 | 0.81 | 0.78 | 0.17 | 0.28 | 0.81 |
| Ratio of family income to poverty | | | | | | | | | |
| <1 | 89/108 (81.1%) | 61/108 (58.3%) | 24/108 (24.5%) | 6/108 (4.9%) | 4/108 (2%) | 38/108 (36.5%) | 5/108 (5.4%) | 7/108 (8.5%) | 1/108 (0.3%) |
| 1-<2 | 160/182 (90%) | 112/182 (64.1%) | 50/182 (25.5%) | 8/182 (5.7%) | 12/182 (5.9%) | 58/182 (33.2%) | 2/182 (0.7%) | 13/182 (7.9%) | 5/182 (4.3%) |
| >=2 | 233/263 (89.4%) | 174/263 (74.2%) | 61/263 (24.5%) | 17/263 (7.3%) | 17/263 (5.3%) | 49/263 (13.1%) | 13/263 (7.9%) | 29/263 (15.1%) | 4/263 (1.3%) |
| P value | 0.25 | 0.024 | 0.97 | 0.68 | 0.36 | <0.0001 | 0.10 | 0.24 | 0.12 |

**Supp. Figure 1**: Study design. NHANES, National Health And Nutrition Examination Series.


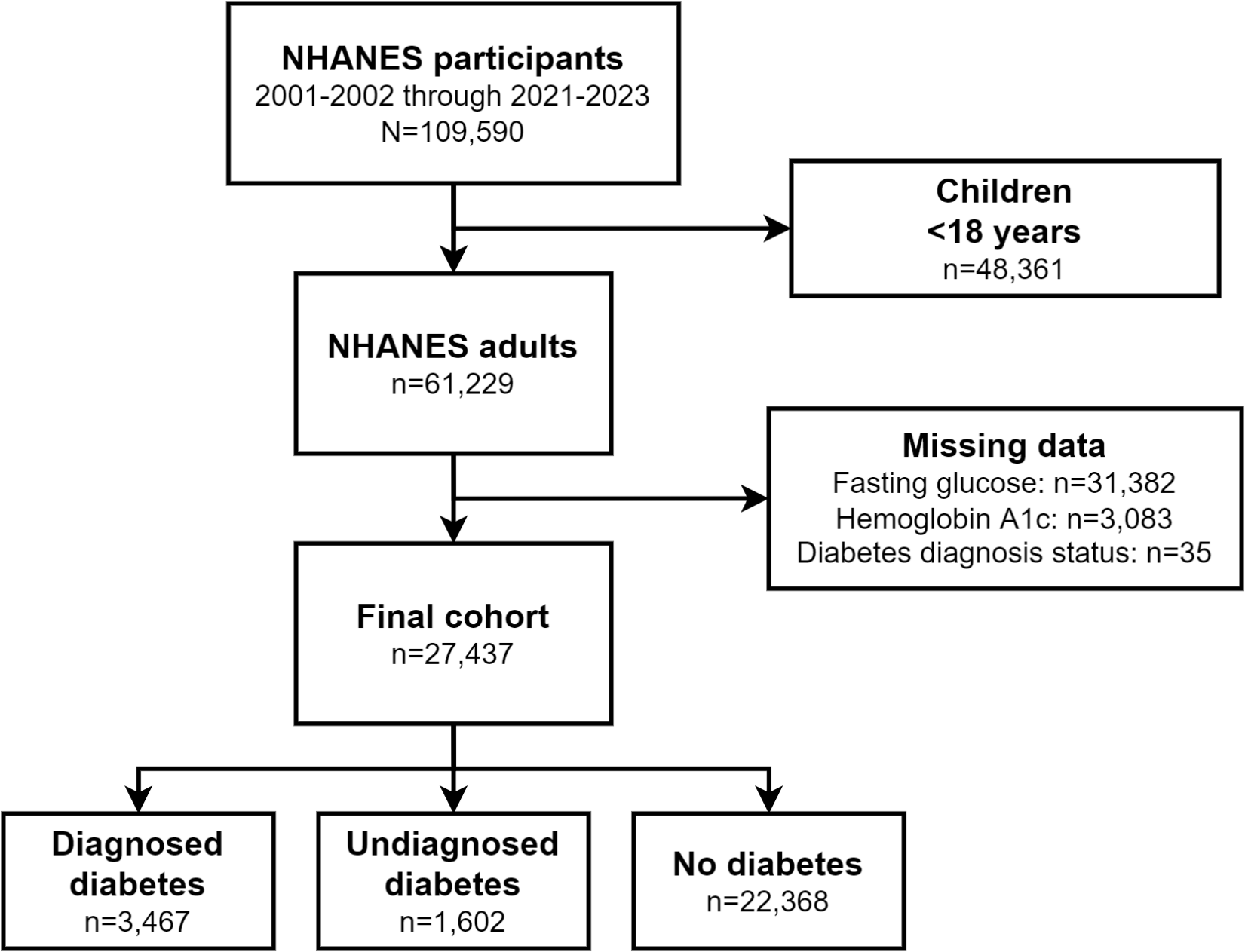


**Supp. Figure 2: Secular trends in glycemic control among adults with diagnosed diabetes**. Prevalence is shown over time in (A) all participants, (B) men, (C) women, (D) Hispanic/Latino participants, (E) non-Hispanic Black participants, and (F) non-Hispanic White participants. Glycemic control is defined as hemoglobin A1c < 8%. P values are based on timepoint analysis set with no joinpoints to identify the overall trend.


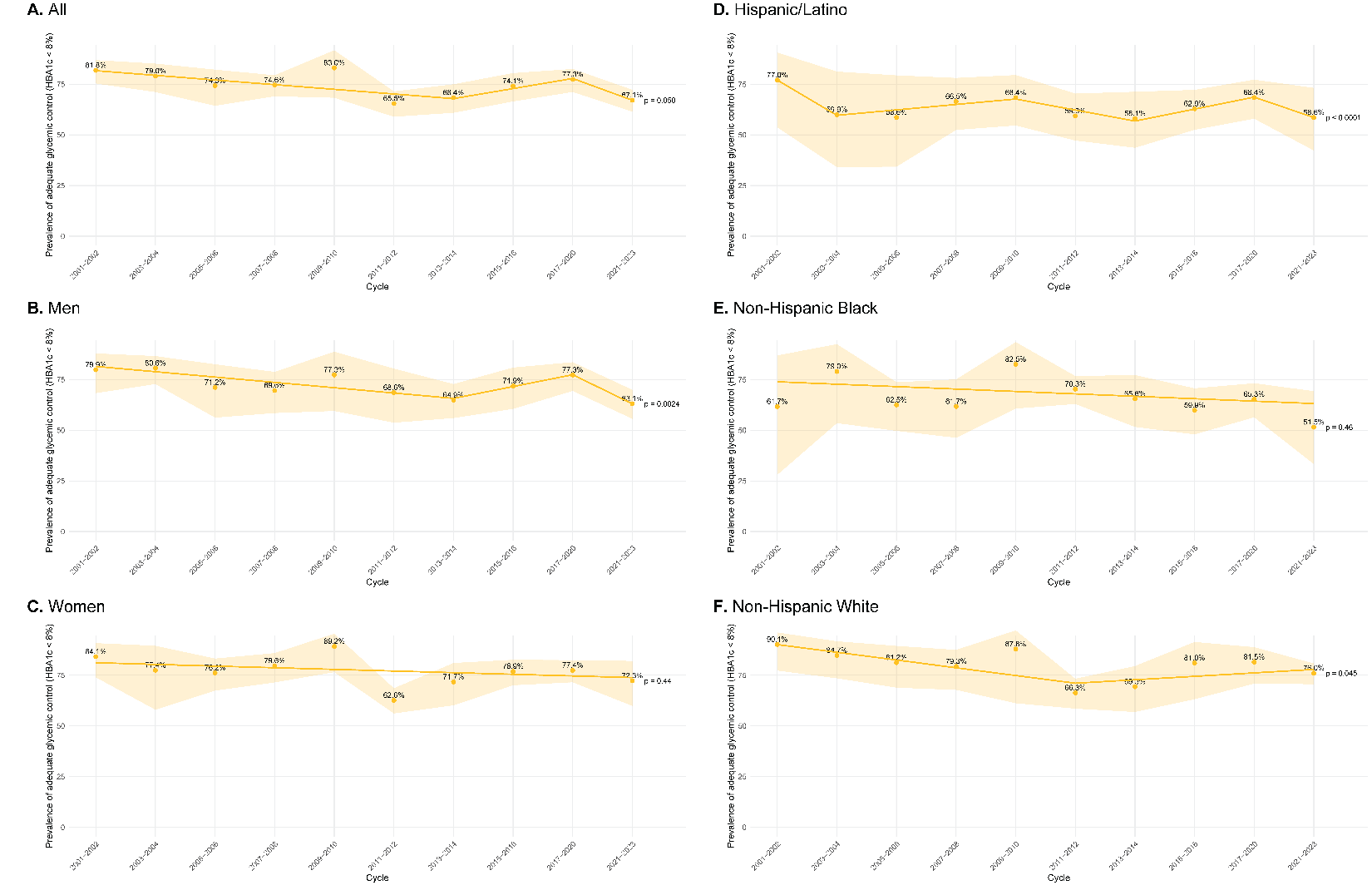

Supplement: Supplementary file 1 — Supplementary file1 (DOCX 265 KB) [file 592_2025_2572_MOESM1_ESM.docx]
